# The Beneficial Effects of Valproic Acid in Thyroid Cancer Are Mediated through Promoting Redifferentiation and Reducing Stemness Level: An *In Vitro* Study

**DOI:** 10.1155/2014/218763

**Published:** 2014-05-15

**Authors:** Vahid Haghpanah, Mohsen Malehmir, Bagher Larijani, Shahin Ahmadian, Kamran Alimoghaddam, Ramin Heshmat, Ardeshir Ghavamzadeh, Khadijeh Adabi, Seyed H. Ghaffari

**Affiliations:** ^1^Endocrinology and Metabolism Research Center, Endocrinology and Metabolism Clinical Sciences Institute, Tehran University of Medical Sciences, P.O. Box 1411413137, Tehran, Iran; ^2^Institute of Biochemistry and Biophysics (IBB), University of Tehran, P.O. Box 13145-1384, Tehran, Iran; ^3^Hematology, Oncology, and Bone Marrow Transplantation Research Center, Tehran University of Medical Sciences, P.O. Box 1411413137, Tehran, Iran

## Abstract

Valproic acid (VPA) has been identified as a histone deacetylase inhibitor, inducing differentiation in transformed cells. However, no study has shown the effect of VPA in the redifferentiation induction and stemness of anaplastic thyroid. The main objective of this study was to evaluate the efficacy of VPA as a differentiation therapy agent in human thyroid cancer based on its effect on stemness and differentiation process. Indications for differentiation of 8305C and B-CPAP cell lines following VPA treatment were obtained by analyzing cell proliferation rate, morphological changes, adherent-dependent colony formation, and Hoechst 33342 staining. The expressions of stemness, differentiation, and aggressiveness specific marker genes were measured by quantitative RT-PCR. VPA treatment effectively showed growth inhibition in both cell lines. The high nuclear-cytoplasmic (N : C) ratio of 8305C cells markedly decreased and treated cells became more epithelial-like. Treated cells showed stronger Hoechst 33342 fluorescence compared with control cells. The hTERT and OCT-4 reduction was paralleled with adherent-dependent colony formation decrement in both cell lines. VPA effectively induced NIS and TTF-1 in anaplastic cells, it whereas showed no clear pattern in papillary cell line. VPA treatment also resulted in the reduction of MMP-2 and MMP-9. These finding suggest that VPA could redifferentiate the anaplastic thyroid cancer cells.

## 1. Introduction

Treatment of advanced thyroid cancer with follicular cell origin, mainly radioactive resistant papillary thyroid cancer (PTC), and iodine/chemotherapy resistant anaplastic thyroid carcinoma (ATC) is notoriously challenging [1, 2]. This is partly because these malignancies have lost the ability to take up radioactive iodine or have dedifferentiated to more aggressive types, making treatments ineffective [2]. According to multistep and fetal cell carcinogenesis models, thyroid carcinoma originates from well-differentiated normal thyroid follicular cells [3], as a consequence of multiple mutations accumulated throughout the entire life span, or as a remnant of fetal thyroid cells [4]. However, recent work has argued that thyroid cancer, parallel to other solid tumors, might originate from thyroid cancer stem cells [5–7].

Thyroid cancer stem-like or stem cells are undifferentiated cells which do not express* NIS* and thus cannot absorb radioactive iodine by the same manner that well-differentiated thyroid cells do. Conventional modalities for thyroid cancer, surgery, radiotherapy, and chemotherapy do not appear to be effective in the targeting tumor-initiating cells; therefore, an alternative therapeutic plan targeting stem-like or stem cells is required to effectively target cancer stem cells. Differentiation therapy which includes methods and therapeutic strategies aiming to force the cancer cells to resume the process of maturation seems promising [8].

Histone deacetylase (HDAC) inhibitors have recently been an object of interest to clinicians for their potential use in cancer therapy as antiproliferative agents exerting their influence by inducing apoptosis, promoting differentiation, and arresting cell cycle [9]. VPA, an established drug in the long-term therapy of epilepsy, has been suggested as a potential agent for differentiation of mesenchymal stem cells [10] and a number of tumors such as glioblastoma [11], head and neck cancer [12], and uveal melanoma [13].

Here, to attribute the redifferentiation and aggressiveness reduction responsible for therapeutic role of VPA in thyroid carcinoma cell lines, we treated ATC (8305C) and PTC (B-CPAP) cell lines with various doses of VPA and monitored the responses by means of cell proliferation, N : C ratio and morphological analysis, adherent-dependent colony formation assay, quantitative RT-PCR, and Hoechst 33342 staining.

## 2. Materials and Methods

### 2.1. Cell Lines and VPA Treatment

Two human thyroid carcinoma cell lines used in this study, B-CPAP and 8305C, were purchased from the DSMZ (Braunschweig, Germany). These cell lines were cultured, as previously described [14]. VPA (Sigma-Aldrich) was diluted at desired concentration in the cell culture medium and treated with 0.1, 1, 5, and 10 mmol/L. Equal volume of DMSO was added to the control samples, in which the final concentration of DMSO did not exceed more than 0.1% of the final medium.

### 2.2. Proliferation Assay (MTT)

Cell proliferation was performed to calculate the appropriate dose for further assays. Briefly, 5000 cells were cultured in a 96-well plate and treated for 96 h with VPA. Then, the MTT solution was added with final concentration of 5 mg/mL. Medium was aspirated after 4 h of incubation, and to solubilize the formazan crystals 100 *μ*L DMSO was added. VPA growth inhibition rate was calculated using the following equation: inhibition rate (%) = optical density (OD)_obs_/optical density (OD)_con_ × 100 in which OD_obs_ and OD_con_ represent the optical densitometry of treated and untreated control (DMSO) cells, respectively.

### 2.3. N : C Ratio and Morphological Analysis

The 8305C cell line was cultured in a 6-well plate and was treated the day after. Briefly, cultured cells were treated with 0.1 and 1 mmol/L of VPA for 96 h and were fixed in 0.1 M phosphate buffered saline (PBS), 2.5% glutaraldehyde (pH 7.4). The N : C ratio and morphological studies were performed under light microscope after Wright-Giemsa (Sigma-Aldrich, Germany) staining, according to the manufacturer instructions. The stained blue to violet area was defined as cytoplasm; in contrast, the stained red to pink area was defined as nucleus. Image J software (NIH Image, Bethesda, MD) was used to analyze the N : C ratio changes. The N : C ratio was derived from the N : C ratio = Ave_nuc_/Ave_cyt_ × 100 relationship, in which Ave_nuc_ and Ave_cyt_ show mean areas of the nucleus and cytoplasm. Stained cells were studied for morphological changes with light microscopy (×40).

### 2.4. Adherent-Dependent Colony Formation

To evaluate the colony formation ability of the 8305C and B-CPAP cells following 0.1 and 1 mmol/L VPA treatment, adherent-dependent colony formation assay was carried out. The colony-forming efficiency (CFE) of each cell line was obtained as CFE (%) = number of colonies/initial seeding density × 100. The data represent the number of colony forming cells in each well after initial seeding.

### 2.5. RNA Extraction and Quantitative Real-Time PCR

Total RNA was extracted from VPA treated cell lines by using High Pure RNA Isolation Kit (Roche, Indianapolis, IN, USA). RNA (1 *μ*g) was reverse transcribed with Prime Script RT reagent kit (Takara), using random hexamer and oligo(dT) primers. Expression of mRNAs was measured by quantitative real-time PCR using StepOnePlus (Applied Biosystem, USA) instrument using SYBER green PrecisionTM 2X qPCRMastermix (PrimerDesign Ltd., UK). Reaction mixture included the following: SYBER green master mix (10 *μ*L), cDNA (2 *μ*L), forward and reverse primers (10 picomol), and nuclease free water (7 *μ*L) which were added in a final volume of 20 *μ*L. Thermocycling included a single initial heat inactivation and denaturation incubation at 95°C for 10 minutes, followed by 40 cycles of 95°C for 5 s and a combined annealing/extension step for 30 s at 60°C. Hypoxanthine phosphoribosyltransferase 1 (HPRT) was amplified as normalizer, and relative-fold differences of target were calculated using the 2^−ΔΔCt^ method normalized to HPRT levels. Primers sequences are indicated in Table 1.

### 2.6. Hoechst 33342 Staining

The dilution of single 8305C cells was cultured on the surface of the glass coverslip in 6-well plates and treated with 0.1 and 1 mmol/L of VPA for 96 h. The medium was aspirated and cells were washed twice with PBS pH 7.4 solution. In order to permeabilize the cells, cold (−20°C) 100% methanol was added and left for 15 minutes in room temperature and rinsed thoroughly three times with PBS. Fresh dilution of the Hoechst 33342 stock solution was added with the final concentration of 1 *μ*g/mL, incubated at 37°C for 15 minutes, and protected from light. The stained cells were immediately examined by IX70 fluorescence microscope (Olympus Optical, London, UK) using blue filter and photographed (excitation, 350 nm; emission, 450 nm).

### 2.7. Statistical Analysis

Experimental data are expressed by mean ± standard deviation of three independent assays for MTT, qRT-PCR, and duplicate for colony formation assay for both cell lines. An independent *t*-test was conducted for comparison between doses. Statistical significance was calculated using paired two-tailed Student's *t*-tests. Statistically different values were defined as significant at *P < 0.05, **P < 0.01, ***P < 0.001. Statistically different values for comparison between 8305C and B-CPAP cells were defined as significant at ^+^ *P* < 0.05, ^++^ *P* < 0.01, ^+++^ *P* < 0.001.

## 3. Results

### 3.1. Effect of VPA on Cell Growth

**Figure 1:**
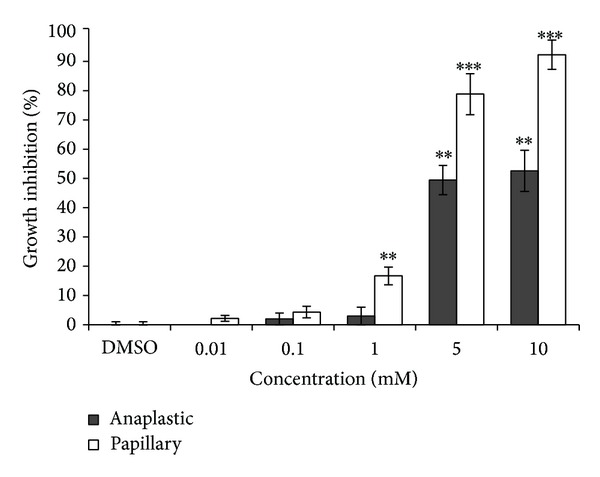
Effects of 0.01, 0.1, 1, 5, and 10 mmol/L VPA treatment on 8305C and B-CPAP cell growth inhibition (GI). Proliferation was measured with an MTT test after 96 h of proliferation in the presence of appropriate amount of DMSO as control or desired VPA. Data are presented as mean of three experiments ± SD. Statistical significance was defined at *P < 0.05, **P < 0.01, ***P < 0.001 compared to the corresponding control.

In this study we used MTT assay to determine the cell growth inhibition and also calculate the appropriate doses for the next assays. As shown in Figure 1, VPA treatment showed a 2% to 52% inhibition in the 8305C cells and 2% to 92% in the B-CPAP cells. Since we aimed to evaluate the redifferentiation effect of VPA low doses, 0.1 and 1 mmol/L which are therapeutically achievable in patient, these appropriate doses with low proliferation inhibition were selected for the next assays.

### 3.2. Significant Morphological Changes Accompanied with N : C Ratio Decrement

**Figure 2:**
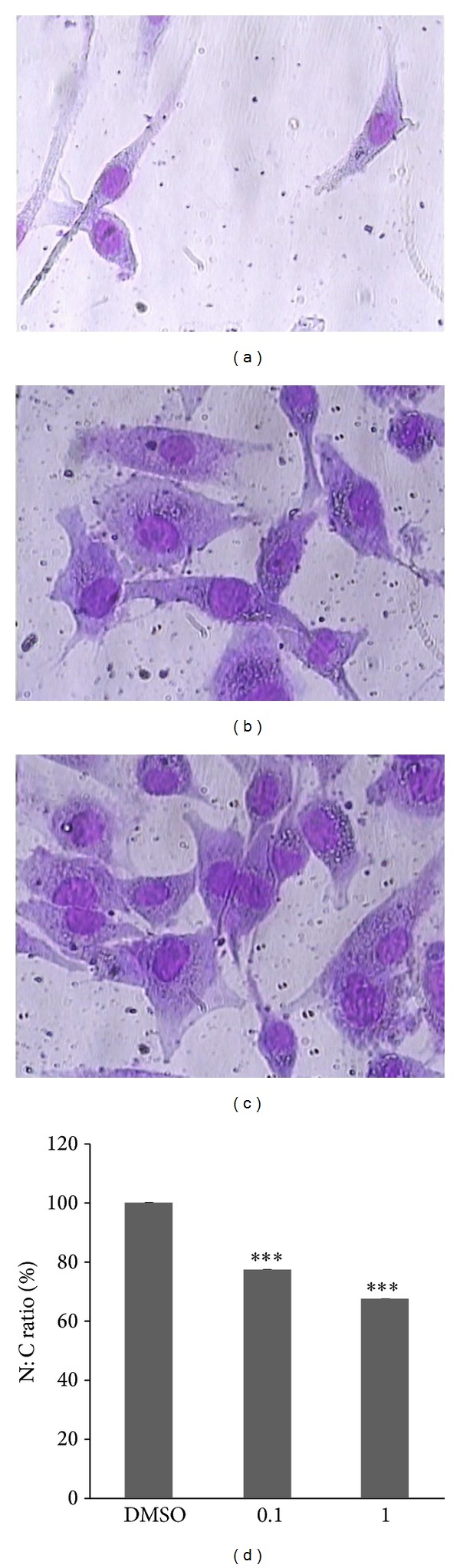
Morphological changes and N : C ratio analysis of 8305C cells following 0.1 and 1 mmol/L VPA treatment for 96 h. (a) Untreated control cells (DMSO) showed spindle and mesenchymal morphology. (b) and (c) 0.1 and 1 mmol/L VPA treated cells showed oval-round to polygonal morphology (magnification 40x). (d) High N : C ratio of the untreated cells decreased dramatically in VPA treated cells. Data are shown as mean ± SD. ***P < 0.001.

N : C ratio measurement was carried out to investigate the beneficial effect of VPA in redifferentiation induction of 8305C cells. The morphology of Wright-Giemsa stained cells showed that the control cells had spindle shape morphology (Figure 2(a)). In contrast, 0.1 and 1 mmol/L VPA treated cells (Figures 2(b) and 2(c)) were oval-round to polygonal. The results of the study revealed that high N : C ratio of 8305C cells reduced following VPA treatment. As shown in Figure 2(d), the N : C ratio of the cells reduced to 77% (*P* < 0.0001) and 67% (*P* < 0.0001) compared to the untreated control (DMSO) in 0.1 and 1 mmol/L treated cells, respectively. In addition, the cells became larger following VPA treatment. Furthermore, mesenchymal morphology of the cells became more epithelial-like after treatment.

### 3.3. VPA Suppressed the Colony Forming Capacity

**Figure 3:**
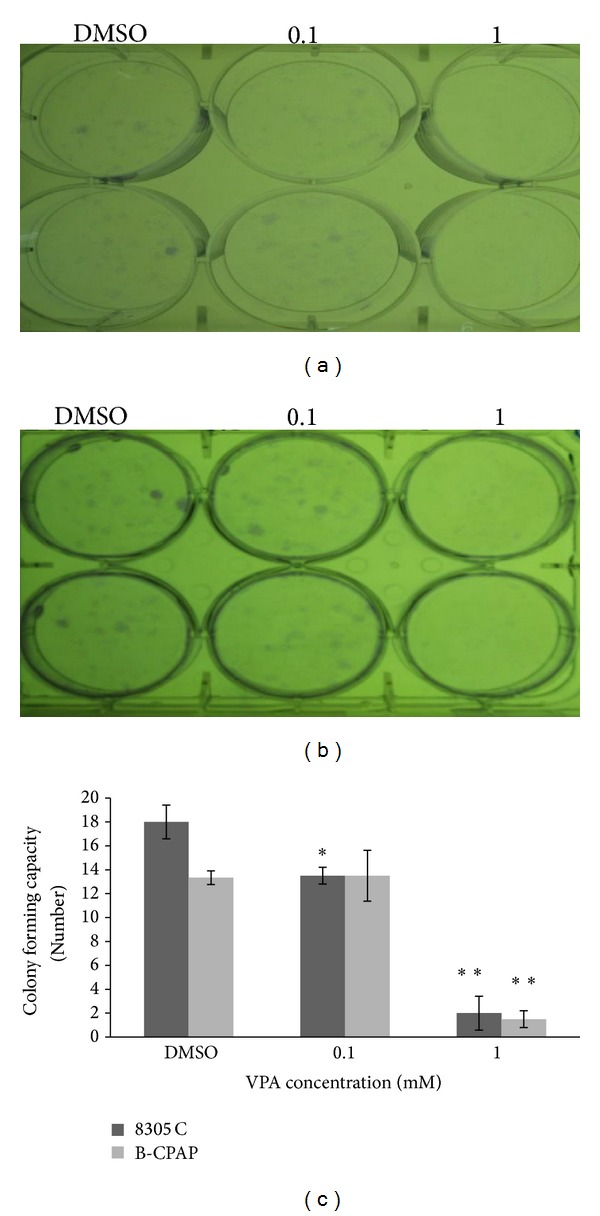
Colony forming capacity of the cells after VPA treatment. Papillary (a) and anaplastic (b) cells were seeded in 6-well plate, and after attachment for 3 h, cells were treated with 0.1 and 1 mmol/L of VPA for 2 weeks. After 2 weeks colonies were fixed with glutaraldehyde (6.0%, v/v), stained with crystal violet (0.5% w/v) and the number of colonies with at least 50 cells was counted. (c) Each data is expressed as mean of colony forming cells ± SD. Statistically different values of *P < 0.05, **P < 0.01, and ***P < 0.001 were determined significantly compared with the corresponding control.

To evaluate the number of the colony forming cells, mainly attributed to progenitor or stem cells, we performed anchorage-dependent colony forming assay. As shown in Figure 3, VPA reduced the colony forming ability of the two cell lines. CFE in 6-well plate in the absence of VPA ranged from 2.7% for B-CPAP cells to 3.6% for 8305C cells. VPA at 0.1 and 1 mmol/L exhibited 25% and 89% inhibition of anchorage-dependent growth in 8305C and also 4% and 89% in B-CPAP cell line, respectively. These results demonstrated that VPA was able to hinder the anchorage-dependent growth of 8305C and B-CPAP cells, indicating a persistent downregulation of growth in the presence of VPA.

### 3.4. VPA-Induced Thyroid Differentiation Marker Accompanied with Downregulation of Stemness and Invasion Related Genes

**Figure 4:**
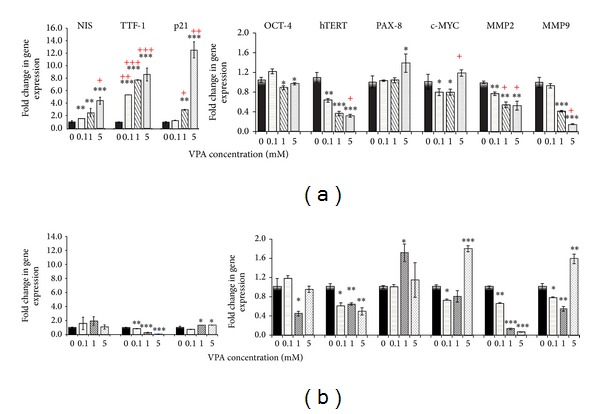
Effect of 96 h VPA treatment on mRNA transcriptional level. The relative mRNA expression of* NIS*,* TTF1*,* p*21,* OCT-4*,* hTERT*,* PAX8*,* c-MYC*,* MMP2*, and* MMP9* was measured following VPA treatment. The relative mRNA expression of each gene was measured by qRT-PCR in the 0.1, 1, and 5 mmol/L VPA treated 8305C (a) and B-CPAP (b) cell lines as described in Section 2. Data are presented as mean ± SD. *P < 0.05, **P < 0.01, and ***P < 0.001. + two cell lines compared to each other. Statistical significance was calculated using paired two-tailed Student's *t*-tests.

In this study we evaluated the effect of VPA on the expression pattern of stem cell markers,* OCT-4* and* hTERT*; thyroid-specific differentiation markers,* NIS*,* TTF1*, and* PAX8*; invasion related markers,* MMP2* and* MMP9*, as categorized in Table 2, by using quantitative real-time PCR. VPA treatment, as shown in Figure 4, decreased the expression of* OCT-4* and* hTERT *in the 8305C (Figure 4(a)) and B-CPAP (Figure 4(b)) cells, with marked reduction in the 8305C cells. The downregulation of* c-MYC*, the main positive regulator of the* hTERT*, correlated with the expression of* hTERT* in the 8305C cells. The expression of thyroid-specific differentiation markers* NIS*,* TTF1*, and* PAX8* significantly induced in the 8305C cell lines. The B-CPAP, however, showed no changes in* NIS* and decreases in* TTF1 *expression. The invasive related* MMP2* and* MMP9* gene expression was reduced in the 8305C cell line in a dose-dependent manner, and also MMP2 expression was reduced in the B-CPAP cell line. Although MMP9 expression was reduced in low doses in B-CPAP cell line, increased expression was observed in the highest concentration.

### 3.5. Brighter Hoechst 33342 Blue Fluorescence in VPA-Treated Cells

**Figure 5:**
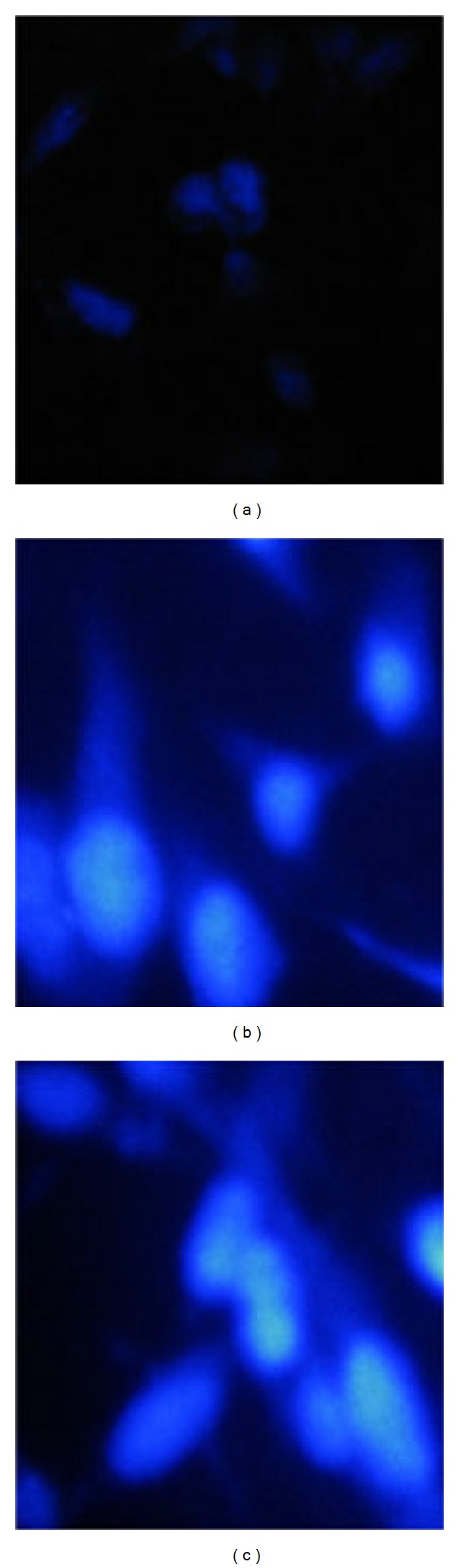
Hoechst 33342 staining of untreated control cells (a) versus 0.1 mmol/L (b) and 1 mmol/L (c) VPA treated 8305C cells. After exposure to VPA for 96 h, fresh dilution of the Hoechst 33342 stock solution was added with the final concentration of 1 *μ*g/mL, incubated at 37°C for 15 minutes, protected from light. The stained cells were examined by IX70 fluorescence microscope using blue filter (excitation, 350 nm; emission, 450 nm) and photographed (magnification 40x).

To determine whether aforementioned observations following VPA treatment are pertinent to the stemness of anaplastic cells, that is, whether redifferentiation affects the Hoechst 33342 accumulation attributed to stem-like characteristics, we analyzed the fluorescence microscopy photographs following VPA treatment. As shown in Figure 5(a), 8305C control cells demonstrated relatively weak and dull staining with Hoechst33342. In contrast, 0.1 and 1 mmol/L VPA treated cells showed a strongly bright and homogenous blue fluorescence (Figures 5(b) and 5(c)). The weak staining of control cells might be explained by high expression of efflux transporter in stem cells, a key property of side population cells, whereas VPA treated cells have lost the efflux ability following redifferentiation.

## 4. Discussion

Different carcinogenesis models have been proposed to describe the cellular origin of thyroid cancer. According to multistep and fetal cell carcinogenesis models, thyroid carcinoma originates from well-differentiated normal thyroid follicular cells [3], or remnant of fetal thyroid cells [4]. Recently, however, research findings support the concept that a small population of thyroid cancer cells displays properties characteristic of stem cells [15–17]. These putative cancer-forming entities are able to drive tumorigenesis; they might also mediate metastasis and are resistant to the effects of chemotherapy and radiation therapy [17]. The resistance of stem cells has important implications for current therapeutic approaches which mainly target the rapidly dividing cells. The implication of the resistance for conventional treatment modalities is that there should be new therapeutic strategies to eradicate tumor initiating subpopulation. Currently, few options are available to treat advanced thyroid cancers, which tend to respond poorly to conventional chemotherapy [18, 19]. Differentiation therapy, therapeutic intervention promoting differentiation rather than killing cancer cells, targets the tumor initiating stem cells and also possesses less toxicities. We focused on the clinically applicable HDAC inhibitor, VPA, as a way to induce redifferentiation of thyroid cancer stem-like or stem cells, because VPA is clinically available and has a well-documented side effects profile. To address the redifferentiation and aggressiveness reduction responsible for observed biological effects following VPA treatment, we monitored the responses of 8305C and B-CPAP cell lines, measuring different variables associated with the stem cells.

Proliferation assay results showed that VPA inhibits the growth of both cell lines, with IC50 of 3 mmol/L and 5 mmol/L for B-CPAP and 8305C, respectively. The inhibition of cell proliferation was accompanied by the increased expression of the* p*21 gene expression (Figures 4(a) and 4(b)). Catalano et al. showed that VPA induces cell-cycle arrest at G1/S by induction of p21 [20]. In our study, expression of p21 was increased dramatically in anaplastic cells and we believe that this might be p53-independent because p53 is mutated in B-CPAP [21] and 8305C [22] cell lines. In addition, the induction of* p*21 in our study is in accordance with other study that VPA, as a class I HDAC inhibitor, activates the transcription of* p*21 by increasing the acetylation of histone H3 and H4 [10]. Brzezinski et al. showed that with advancing tumor grade in thyroid carcinoma, the expression of the p21 is progressively lost [23].

Morphometric analysis, including N : C ratio and cell sizes, has diagnostic and prognostic values by which pathologists evaluate histological grade. As a cell matures and becomes more differentiated, the size of its nucleus generally decreases. For example, “blast” forms of erythrocytes, leukocytes, and megakaryocytes start with an N : C ratio of 4 : 1, which decreases to 2 : 1 or even 1 : 1 as they mature. Although the N : C ratio is fairly constant for a given cell type, an increased N : C ratio is commonly associated with precancerous dysplasia as well as with malignant cells. Anaplastic thyroid cancer is undifferentiated (hence the name), categorized as grade IV with a large N : C ratio. Therefore, the N : C ratio could be exploited as an indicator to evaluate the differentiation status of the cells. According to the results of our study, the N : C ratio of the anaplastic cells reduced markedly after VPA exposure, accompanied by a morphological shift from a spindle shape to an epithelial-like shape in the treated cells. Giant cell and spindle shape cells were rarely observed following VPA treatment. The morphological changes following VPA treatment demonstrated by this study are further supported by the finding of a papillary-like shape of anaplastic thyroid cancer following combination therapy with VPA in a 51-year-old case by Noguchi et al. [24]. We observed that VPA was capable of restoring a differentiated phenotype.

Anaplastic thyroid carcinoma shows a marked epithelial-mesenchymal transition (EMT) [25, 26], generating cell with stem-like characteristic. The loss of a high N : C ratio and mesenchymal phenotype of the cells might suggest that stem cells have been committed to differentiate, losing the characteristic of stemness, which is further supported by downregulation of stem cell markers, as will be described later. The abrogation of EMT transition by HDAC inhibitor, trichostatin, has been shown in hepatocytes [27].

It has been shown that progenitor or stem cells which are enriched with CD133^+^ and* OCT-4 *have higher colony-forming capacity [5], in addition, the expression of* OCT-4 *in dedifferentiated cells leads to cancer stem-like cell features with the acquired ability to form tumor spheroids, increased resistance to chemotherapeutic agents, and increased tumorigenic capacity [28]. We evaluated the percentage of clonogenic cells to define subset cells endowed with colony-forming ability. The result of the colony-forming assay was correlated to the downregulation of* OCT-4 *expression by real-time PCR. The colony-formation assay is considered to be a stringent* in vitro *assay for malignantly transformed cells [17]. Therefore, VPA-induced differentiation could curtail the number of progenitor or stem cells.

The expression of* hTERT* in stem cells endows them with an indefinite cell proliferation, to bypass senescence and be immortal [29]. The stem cell marker* OCT-4 *and* hTERT* downregulation in this study supported the idea that VPA promoted differentiation in undifferentiated cells, which was confirmed further by colony formation and Hoechst 33342 staining. We observed that the expression of two known thyroid transcription factors,* PAX8* and* TTF1* and also* NIS *increased after VPA treatment in 8305C cells. Interestingly, however,* TTF1* showed a reduction and* PAX8* and* NIS *exhibited no clear pattern in B-CPAP cells. Downregulation of* TTF1* and* NIS* expression in B-CPAP cells following HDAC inhibitors has been reported by Puppin et al. [30]. Our results being in line with those obtained by Fröhlich et al., which reported that VPA could not induce thyroid differentiation marker,* NIS*, in B-CPAP cell line [31]. The fact that re-differentiation with VPA seems capable of inducing the expression of the* NIS* gene is particularly intriguing because restoration of* NIS* activity could render less differentiated thyroid carcinomas amenable to radioiodine therapy. The result of our study gives further credence to the notion that anaplastic thyroid cancer might originate from cancer stem-like or stem cell which is supported by monitoring the expression pattern of the both cell lines after treatment. Expression pattern indicated that anaplastic cells, which show more stem cell related characteristics, followed an expected pattern by downregulation of stem cell markers,* hTERT* and* OCT-4*, and induction of thyroid differentiation markers,* NIS*,* TTF1*, and* PAX8*. In contrast, papillary cells showed no clear pattern relevant to re-differentiation following VPA treatment. VPA demonstrated some selectivity for stem-like anaplastic cells and less effect on differentiated papillary cells.

The side population cells that exclude Hoechst 33342 dye are enriched in stem cells and progenitor cells with relatively weak staining when cells were stained with Hoechst 33342. The result of our study shows that 8305C cells showed weak staining with Hoechst 33342 dye. In contrast, the VPA treated cells exhibited bright and homogenous staining compared to the control cells. This might imply that the stem cells have lost their ability to efflux Hoechst 33342 staining following redifferentiation induced by VPA. The result of Hoechst staining in our study is in accordance with another study that stem cells derived from human goiter, isolated as a side population by Hoechst 33342 efflux ability, displayed a high N : C ratio and expressed the stem-cell marker* OCT-4 *[32].

Furthermore, it has been proposed that thyroid cancer stem cells might mediate metastasis, which remains the predominant cause of lethality in patients with thyroid cancer [17]. It is possible that approaches targeting stem cells could modulate invasive behavior of anaplastic thyroid carcinoma, a critical step in the clinical management of this disease. A growing amount of experimental evidence indicates that matrix metalloproteinases (MMPs) family members are involved in almost all metastatic steps [33]. The B-CPAP and 8305C cells have increased, compared to normal thyrocytes, the expression of* MMP2* and* MMP9*, mediating the aggressive behavior in the both cell lines. Our study provides evidence supporting a role for VPA in suppressing aggressiveness through alteration of genes directly involved in metastasis. This could be achieved by transcriptional repression of key MMPs, MMP2 and MMP9.

Clinical trials for the use of VPA in the treatment of malignant tumors are already under way; however, its mechanism in anaplastic thyroid cancer, which might originate from thyroid cancer stem cells, is poorly understood. This study shows that VPA is effective on ATC and presumably reduction of aggressiveness and stem cell markers are the underlying mechanisms of VPA effect on anaplastic thyroid carcinoma, suggesting that reduction of aggressiveness and stemness may be a potential therapeutic target for undifferentiated and/or cancer stem cells. The biological behavior of cell lines does not always reproduce the biological behavior of the real tumor. Therefore, further* in vivo* and clinical studies, since the VPA is clinically applicable, are needed to determine the clinical efficacy of VPA on invariably lethal anaplastic thyroid carcinoma.

## Figures and Tables

**Table 1:** Primer sequences for quantitative RT-PCR.

Gene	Accession number	Forward primer (5′-3′)	Reverse primer (5′-3′)	Size (bp)
HPRT	NM_000194	TGGACAGGACTGAACGTCTTG	CCAGCAGGTCAGCAAAGAATTTA	111
NIS	NM_000453	TGCGGGACTTTGCAGTACATT	TGCAGATAATTCCGGTGGACA	133
TTF-1	NM_001079668	AGCACACGACTCCGTTCTC	GCCCACTTTCTTGTAGCTTTCC	68
p21	NM_000389	CCTGTCACTGTCTTGTACCCT	GCGTTTGGAGTGGTAGAAATCT	130
OCT-4	NM_001173531	CTTGAATCCCGAATGGAAAGGG	GTGTATATCCCAGGGTGATCCTC	164
hTERT	NM_001193376	AACCTTCCTCAGCTATGCCC	GCGTGAAACCTGTACGCCT	210
PAX-8	NM_003466	TGGGGACTACAAACGCCAGA	GCTGTCCATAGGGAGGTTGAAT	163
c-MYC	NM_002467	CCACAGCAAACCTCCTCACAG	GCAGGATAGTCCTTCCGAGTG	105
MMP-2	NM_004530	CTTCCAAGTCTGGAGCGATGT	TACCGTCAAAGGGGTATCCAT	119
MMP-9	NM_004994	GGGACGCAGACATCGTCATC	TCGTCATCGTCGAAATGGGC	139

HPRT: Hypoxanthine phosphoribosyltransferase 1; NIS: sodium/iodide symporter; TTF1: thyroid transcription factor 1; p21: CIP1/WAF1; OCT-4: octamer-binding transcription factor 4; hTERT: human telomerase reverse transcriptase; PAX8: Paired box gene 8; c-MYC: v-myc myelocytomatosis viral oncogene homolog; MMP-2: matrix metallopeptidase 2; MMP-9: matrix metallopeptidase 9.

**Table 2:** List of genes that were used and categorized based on their biological function.

Biological function	List of genes
Stem cell marker	OCT-4, hTERT
Thyroid differentiation marker	NIS
Thyroid transcription factor	PAX-8, TTF-1
Invasion	MMP-2, MMP-9
Cell cycle	p21
Multifunctional transcription factor	c-MYC
